# Perceptions of prevalence and management of post-acute sequelae of SARS-CoV-2 (PASC) infection among healthcare workers in Kweneng District, Botswana: Report of a district-wide survey

**DOI:** 10.1371/journal.pgph.0003865

**Published:** 2024-11-27

**Authors:** Tebogo T. Mamalelala, Savannah Karmen-Tuohy, Lettie Chimbwete, Ditebogo J. Mokone, Roger Shapiro, Claire Young, Sara Schwanke Khilji

**Affiliations:** 1 Faculty of Health Sciences, School of Nursing, University of Botswana, Gaborone, Botswana; 2 NYU Grossman School of Medicine, New York University, New York, New York, United States of America; 3 Department of Health Sciences, University of Pretoria, Pretoria, South Africa; 4 Ministry of Health, Gaborone, Botswana; 5 Department of Immunology and Infectious Diseases, Harvard TH Chan School of Public Health, Boston, Massachusetts, United States of America; 6 Botswana-Harvard Health Partnership, Gaborone, Botswana; 7 Department of Medicine, Oregon Health & Science University, Portland, Oregon, United States of America; University of California, UNITED STATES OF AMERICA

## Abstract

Over 9.5 million confirmed cases of COVID-19 infection have been recorded in Africa. The syndrome of post-acute sequelae of SARS-CoV-2 infection (PASC) affects an estimated 32% to 87% of COVID patients globally. Data regarding prevalence and impact of PASC in Botswana are limited. This study used a cross-sectional survey design to query healthcare workers in Kweneng District, Botswana about perceived PASC prevalence, duration, symptoms, impact, and management strategies. The survey was disseminated to participants via pre-existing WhatsApp groups and paper copy. Descriptive statistics were used to analyse quantitative data, including demographic data. 72 respondents consented and completed the survey, from an estimated 650 staff meeting eligibility criteria; 63% were female and 36% were male. The majority (90%) were nurses, with doctors and “other” accounting for 6% and 4% of respondents, respectively; no administrators responded. Over half (72%) worked at primary care facilities and 28% worked in hospitals. Nearly all (93%) indicated seeing patients with PASC on a weekly basis, though the majority (61%) identified these patients as comprising <10% of total patients. The most frequently reported PASC symptom was persistent cough (64%), followed by shortness of breath (54%) and fatigue (49%). A substantial minority of respondents were unsure how to manage common PASC symptoms, with 29% and 36% indicating uncertainty regarding management of persistent cough and fatigue, respectively. Findings indicate that PASC symptoms are frequently encountered in clinical practice in Botswana with significant overlap with acute COVID-19, influenza-like illnesses, and tuberculosis, likely placing increased burden on existing health system processes. Providers reported uncertainty in managing presumed PASC, and current practice patterns may contribute to unintended adverse effects. Clear clinical algorithms for PASC screening, diagnosis, and management should be developed and disseminated in Botswana to mitigate the effects of PASC symptoms and improve the quality of life of COVID-19 survivors.

## Introduction

As of January 2024, over 770 million confirmed cases of SARS-CoV-2 infection have been identified, with 9.5 million recorded in Africa [1]. The syndrome “post-acute sequelae of SARS-CoV-2 infection,” or PASC, affects an estimated 32% to 87% of patients with SARS-CoV-2 (hereafter referred to as COVID-19) globally, representing a significant emerging disease burden [2]. A growing body of literature demonstrates the public health challenge posed by PASC [3–6]. As COVID-19 infections continue to be identified around the world, healthcare systems globally face the need to address the growing morbidity associated with PASC. In Botswana, an upper-middle income country in Southern Africa, the prevalence and typical presentation of this syndrome remain unknown.

While the clinical definition of PASC is evolving [7,8], the WHO post-COVID-19 working group defines PASC as the persistence or development of new symptoms three months after the first SARS-CoV-2 infection that last for at least two months without an alternative etiology identified [9]. Commonly cited post-acute symptoms of COVID-19 infection include chronic fatigue, shortness of breath, and cognitive impairment [10–12]. Similarly, a systematic review of long COVID in African populations, which included 14 studies from seven African countries, reported that fatigue, shortness of breath, and confusion or lack of concentration were the most common symptoms, with prevalence rates of 41%, 25%, and 40%, respectively [13]. Several prospective cohorts of PASC have been documented, each with unique case definition, study population size and composition, set of assessed symptoms, and follow-up periodicity and duration [9,14]. A globally standardised definition is necessary to progress in characterising PASC epidemiology and developing prospective treatments for this syndrome [9].

Botswana recorded its first case of COVID-19 infection in March 2020 [15], with a total of 330,368 verified cases reported to date [16]. However, the incidence and prevalence of PASC in Botswana have not yet been defined in the published literature. Data are similarly limited to describe the current prevalence and impact of PASC in Kweneng District, Botswana. Further, given the novelty of the PASC syndrome, healthcare workers (HCWs) in Kweneng District have to date received little training about the triage and management of these symptoms. Current lack of epidemiologic data and clinical management guidelines represent important gaps in the existing evidence to inform a systematic response to PASC in Botswana.

Kweneng District was selected as the study site due to an existing collaboration between district health system staff and researchers from the Botswana-Harvard Health Partnership. The collaboration focuses on clinical education, quality improvement, and related research with topics of study determined in close dialogue with partners in Kweneng District. The study was conceived by district hospital nursing leadership in response to the observed growing impact of PASC on hospital healthcare workers, both personally and professionally.

This study aims to explore the perceived prevalence of PASC and related clinical management practices in Kweneng District as reported by district-level HCWs and healthcare leaders, in order to inform locally relevant recommendations for PASC management. The primary objective is to describe HCW’s perceptions of PASC burden among patients seeking care at government health facilities across Kweneng District. The secondary objectives of the study are to: (1) identify common presenting symptoms of PASC as perceived by HCWs; (2) identify management approaches for common PASC symptoms currently in use by study participants; and (3) assess interest among district HCWs for PASC-related clinical resources and support interventions. In this report, we present findings from a survey addressing secondary objectives one through three. A second, qualitative component utilising key informant interviews to obtain more nuanced data relevant to secondary objectives two and three was completed following the survey; these results will be published separately. The anticipated outcome from the results of these combined studies is the planned development of clinical practice guidelines for the management of common PASC symptoms within the context of government-sponsored health facilities in Kweneng District, Botswana.

## Methods

A cross-sectional survey design was utilised, surveying HCWs about the perceived prevalence of PASC, common presentations of the syndrome, and current management strategies.

### Study setting

The study was conducted in Kweneng District, Botswana, a Ministry of Health district providing care to a population of 387,983 individuals [17]. Kweneng District hosts a 350-bed public hospital, which is located approximately 60km from the country’s capital city and serves the village of Molepolole, one of three highly populated villages in the country (population ~ 75,000) [17]. The hospital receives referrals from a range of health facilities across the district, including a 25-bed primary hospital; a military hospital; 23 primary care clinics; and 38 health posts. As a secondary-care hospital, the public hospital provides specialist services (Pediatrics, Surgery, Obstetrics & Gynecology, and Medicine), laboratory services, pharmaceutical services, and basic radiography (x-ray and ultrasound). The hospital has limited sub-specialist presence and does not have advanced imaging services (e.g. computed tomography [CT] or magnetic resonance imaging [MRI]). Located on the edge of the Kalahari Desert, Kweneng District is expansive and sparsely populated, covering an area roughly the size of Taiwan. Healthcare facilities vary in distance to the referral hospital, ranging from less than a kilometer to 252 km from this facility. Kweneng West reports the highest poverty levels in Botswana at over 50%, with unemployment averaging 11.8% for males and 9.7% for females [17].

### Survey development

A 25-item survey was developed by members of the research team, in consultation with other Kweneng District HCWs. Survey questions were informed by the extant published literature on PASC, as detailed in the Background section, then reviewed by members of the study team to establish face validity. The survey tool was initially developed using the Google Forms platform and was subsequently converted to a Word document to facilitate paper-based distribution (S1 Text). The survey tool was piloted on six nurses and adjustments were made as indicated to ensure clarity of questions and response options.

### Subject population & recruitment

The target population included HCWs employed in patient-facing roles, including general nurses, nurse practitioners, medical officers (general practitioners), specialists, and district health management team staff working at government health facilities in Kweneng District. Inclusion criteria stipulated that participants be over 18 years old and literate in English (the standard language for medical care provision and documentation in Botswana). Participant recruitment took place 1^st^ August 2022 through 11^th^ February 2023. An electronic version of the survey, constructed on Google Forms, was initially disseminated electronically to HCWs across Kweneng District via pre-existing WhatsApp groups during August 2022, and only 13 respondents completed the survey. At the time of recruitment, an estimated 640 healthcare workers (including 581 nurses, 46 medical officers, six specialist doctors, and several healthcare leaders) were working in the district.

Given low response to the online survey, 80 paper copies of the survey were distributed to a convenience sample of clinics around the district between November 2022 and mid-February 2023. Specifically, study team members distributed paper copies to twenty primary care facilities throughout the main village in Kweneng District, Molepolole; these clinics included the larger primary care clinics typically staffed by both doctors and nurses (as opposed to nurses alone). The number of paper surveys distributed to each primary care facility corresponded to the number of HCWs on duty at that time. The remaining paper copies were allocated to points of entry to the district hospital (e.g. the Accident & Emergency and Outpatient Departments). Under existing referral mechanisms in the government sector, all clinics district-wide are allowed to refer patients to the district hospital utilizing government-supported medical transport, escorted by a HCW from the referring primary care facility. All escorting HCWs were invited to complete the survey. Surveys were conducted anonymously; no directly identifying data were collected from participants. Descriptive statistics were used to analyse quantitative data collected, including demographic data. Categorical variables were expressed as frequencies and percentages.

### Ethics statement

This study was carried out in strict accordance with accepted human subjects research practice. The protocol was reviewed and approved by the Health Research & Development Committee, Health Research Unit, Botswana Ministry of Health (Protocol Number: 00950; Approval Number HPRD: 6/14/1), with a risk determination of “no greater than minimal risk.” Permission to conduct research within Kweneng District was obtained from the Scottish Livingstone Hospital Research Committee prior to initiation of data collection, a committee chaired by the hospital Chief Medical Officer and including representatives from multiple healthcare cadres. All potential survey participants received and were asked to review a Participant Information Sheet prior to agreeing to participate (either paper-based or electronic, according to the mode of survey delivery). To ensure anonymity of survey responses, participants were not asked to provide written consent with a name and signature. Instead, they received the following notification upon completion of Participant Information Sheet review: “By proceeding with this survey, you indicate your consent to participate in the study as described above.”

### Inclusivity in global research

In addition to following standard human subjects research practice as outlined above, the study team endeavoured to demonstrate good practice for global health research, with emphasis on promoting equity in health through study design, dissemination of findings, and inclusion of community interests within the context where the research was conducted. Additional information regarding ethical, scientific, and cultural considerations specific to inclusivity in global research incorporated into the design and conduct of this study can be found in the (S2 Text).

## Results

A total of 79 respondents completed the survey (13 online and 66 via paper survey), representing 12% of eligible HCWs in the district (S1 Data). As snowballing was intentionally encouraged for dissemination via WhatsApp, the total number of recipients of the electronic survey link could not be ascertained; thus, the response rate for the online survey could not be calculated. Instead, the calculated response rate among HCWs receiving a paper copy of the survey was 83%, with 66 of the 80 distributed paper-based surveys completed. Of the total respondents (paper and online), 91% provided informed consent. Results from the seven surveys where informed consent was not explicitly indicated were removed from the analysis. Most respondents (90%) were nurses, with doctors and “other” accounting for 6% and 4% of respondents, respectively, no respondents self-identified as healthcare administrators. Over half (72%) worked at primary care facilities, and 28% worked in hospitals. In terms of gender distribution, 63% of respondents were female and 36% were male (Table 1).

**Table 1:** Demographic characteristics of participants.

	n	%
**Workplace**		
Hospital	20	27.8
Primary Care Facility	52	72.2
**Profession**		
Nurse	65	90.3
Doctor	4	5.6
Other	3	4.2
**Gender**		
Female	45	62.5
Male	26	36.1
No response	1	1.4
**Age**		
21–30	21	29.2
31–40	29	40.3
>40	22	30.5
Total	72	100.0

Survey responses highlight the high burden of PASC symptoms among patients presenting to healthcare facilities across Kweneng District. Almost all respondents (93%) indicated seeing patients with PASC on a weekly basis, though the majority (61%) estimated that these patients accounted for less than 10% of total patients (Table 2). Regarding the typical timing of PASC presentation, measured from time since diagnosis of acute COVID-19 infection to presentation to a healthcare facility with PASC symptoms, roughly half of participants responded either that timing of onset varies or they were unsure; 44% indicated a time to presentation of less than six months.

**Table 2:** Estimated burden of symptoms among patients at government facilities.

		n	%
Number of patients per week treated for symptoms of PASC	0	5	7.0
	1–5	51	78.9
	>10	15	21.1
Proportion of patients seeking treatment for PASC symptoms	Less than 10% of patients	44	61.1
	More than 10% of patients	11	15.3
	Unsure	17	23.6
Time from COVID-19 infection to presentation with PASC symptoms	0–6 months	32	44.4
	Greater than 6 months	5	6.9
	It varies	16	22.2
	Unsure	19	26.4

Respondents were asked to select the five most frequently encountered symptoms in patients suspected to have PASC from a list of common symptoms. They were also offered an opportunity to enter a free-text response for any symptom not listed. The most frequently selected PASC symptom was persistent cough, chosen by 64% (n = 46) of respondents. This was followed by shortness of breath, which was reported as one of the five most common PASC symptoms by 54% (n = 39) of respondents. Other frequently selected symptoms were fatigue (49%), headache (44%), and muscle or body aches (42%). Additional, less frequently selected PASC symptoms, are detailed in Table 3.

**Table 3:** Most common symptom, as reported by respondents.

Symptom	n	%
Persistent cough	46	63.9
Shortness of breath	39	54.2
Fatigue	35	48.6
Headache	32	44.4
Muscle or body aches	30	41.7
Joint pain	28	38.9
Fever	17	23.6
Sore throat	16	22.2
Loss of taste/smell	15	20.8

After selecting the five most frequently encountered PASC symptoms, respondents were asked to rank these selected symptoms from most to least frequently encountered. In response to this question, headache was ranked first as the most frequently encountered PASC symptom by the largest proportion of respondents (22%, n = 16), followed by persistent cough, which was ranked first by 21% (n = 15) of respondents.

The final section of the survey invited participants to complete free-text short answers to indicate typical management of the following common PASC symptoms: persistent cough, persistent shortness of breath, chronic headache, intermittent chest pain, and fatigue. The large majority (89%) of respondents indicated that they routinely treat persistent shortness of breath with corticosteroids (route, i.e. inhaled vs oral, not specified). Similarly, the large majority reported prescribing over-the-counter analgesics for chronic headache (88%). Nearly half of respondents reported prescribing antibiotics for treatment of persistent cough attributed to PASC (Table 4). A substantial proportion of respondents were unsure how to respond to questions regarding managing common PASC symptoms, with 29% and 36% of participants indicating uncertainty regarding management of persistent cough and fatigue, respectively.

**Table 4:** Typical medical management of common symptoms attributed to PASC.

Symptom	N	%
Chronic headache with analgesics	50	92.5
Shortness of breath with corticosteroids	41	89.1
Persistent cough with antibiotics	31	43.7
Fatigue with Vitamin B Complex	20	15.7

The survey concluded with questions assessing participants’ interest in receiving support for PASC management. Almost all participants (92%; n = 66) said they would attend a workplace-based training/informative session focusing on treatment for PASC symptoms, while an additional three respondents were unsure but indicated they might consider this. Additionally, 90% (n = 65) stated they would refer patients with COVID-19-related complications to multidisciplinary services, including physiotherapy, occupational therapy, and mental health services, if available; an additional three also expressed uncertainty about utilizing such services but indicated they might consider this possibility.

## Discussion

### PASC burden and characterization in Botswana

Findings from this study indicate that the large majority of HCWs in Kweneng District encounter patients presenting with symptoms consistent with PASC on a weekly basis. While PASC prevalence cannot be estimated from the survey responses, the reported frequency of patient encounters strongly suggests that PASC is prevalent in Botswana and that patients are seeking care at various levels of the public healthcare system. For regional context, epidemiological studies conducted in Africa have reported PASC prevalence estimates at two months post-infection ranging from 35% (South Africa) [18] to 51% (Zambia) [19] with studies from Nigeria and Morocco reporting 41% [20] and 47% respectively [21]. Prevalence of PASC symptoms differs according to severity of initial COVID-19 infection, with a South African study finding that 46.7% of patients hospitalised for COVID-19 and 18.5% of nonhospitalized patients experienced ≥ 1 symptoms at six months post-infection [22]. Official reports may underestimate the real PASC burden in Southern Africa; Mendelsohn *et al*. found that only 24% of South African patients self-reporting symptoms consulted a clinician for long COVID, and only 7% received care for PASC in the public sector [18]. To the best of our knowledge, no prevalence study has been conducted to date in Botswana to confirm whether PASC prevalence is similar across the subregion.

The need for targeted studies of the prevalence and impact of PASC in Botswana is further underscored by typical risk factors for PASC observed in similar populations. Factors associated with increased risk for development of PASC include older age, female sex, multiple comorbidities, and severe acute COVID-19 infection [23,24]. Notably, PASC burden in the region appears to vary according to baseline health. For instance, a regression analysis of observational data from Ghana found that patients with hypertension and diabetes mellitus had four times the odds of developing long COVID compared to those without comorbidities [25]. Karuna and colleagues conducted a multi-country observational cohort study of post-COVID conditions, finding that patients with a history of lung disease reported 45–58% longer duration of general, neurologic and respiratory symptoms [26]. These findings are highly relevant to the context in Kweneng District and Botswana as a whole, where combined death and disability due to diabetes increased over 40% from 2009 to 2019 (now ranking in the top ten causes of combined death and disability in the country), while hypertension rose to the fourth leading risk factor driving death and disability over the same time period [27]. Additionally, many people living in Kweneng District suffer from chronic lung disease related to high rates of background pneumoconioses, often linked to occupational exposures (particularly mining) [28] and complicated by high tuberculosis incidence, estimated at 235 per 100K population in 2021 [29].

Responses to this survey provide insight into typical patterns of PASC presentation in Botswana from the perspective of front-line HCWs in the public sector. The most frequently reported presenting PASC symptoms in Kweneng District—persistent cough, shortness of breath, fatigue, headache, and muscle or body aches—are similar to those reported in studies in the Southern African subregion, as well as globally. Findings from our study overlapped considerably with those reported by Pretorius *et al* [30], conducted in South Africa; this study found that the most commonly reported PASC symptoms locally were fatigue, brain fog, loss of concentration and forgetfulness, shortness of breath, joint and muscle pains. Fatigue was shown to be the most prevalent symptom and the primary cause for patients to seek medical attention across Africa [31,32].

The emergence of headache as the most frequently cited top PASC symptom in this study is notable, as this represents a potential shift in epidemiology of a common condition within the Botswana healthcare context. Chronic headache was a common indication for referral for specialist review in Botswana even prior to the onset of the COVID-19 epidemic, placing significant burden on a primary care system in which the per capita density of doctors (general doctors as well as specialists) is only 3.8 per 10,000 [33]. Differentiating PASC-related headache from other aetiologies of chronic headache is important to avoid placing additional stress on existing referral processes within the public sector and highlights the need for a clear definition of PASC for use by primary care practitioners within Botswana. Additionally, there may be a synergistic effect on patients with prior history of chronic headache before known infection with COVID-19, as the infection has been observed to exacerbate the symptoms of individuals who already had headaches before getting COVID-19 [34]. Chronic headaches attributed to prior COVID-19 infection have been shown to have a substantial negative influence on social and psychological activities; therefore, prompt and comprehensive management of these sequelae should be a priority [35]. Results from the present study reveal that chronic headache following COVID-19 infection is managed primarily with analgesia alone in Kweneng District, suggesting potential for improvement through development of multidisciplinary care processes for patients suffering from chronic headache and other PASC symptoms.

Interpretation of these results regarding the most common presenting symptoms of suspected PASC must consider the current lack of a clear, consistent case definition in use in the study context. All the reported common symptoms were frequently encountered in isolation in primary care settings across Kweneng District even prior to the advent of the COVID-19 pandemic. As such, primary HCWs—particularly nurses, who are the first point of contact for the majority of patients presenting to primary care facilities in Botswana—have established mental frameworks for diagnosing and providing basic management for these common complaints. In the absence of a common case definition for PASC, we suspect that attribution of these symptoms to possible PASC by study participants occurs in cases where typical treatment algorithms were unsuccessful. This is an area requiring further investigation to substantiate.

### Uncertainty regarding PASC management

Survey results suggest that HCWs at various health system levels across Kweneng District lack confidence in managing common PASC symptoms, with approximately one-third of respondents indicating uncertainty regarding management of frequently encountered PASC symptoms (persistent cough, fatigue). Among respondents who completed free text responses detailing current practices for managing common PASC symptoms, most reported using management strategies more appropriate in the context of treating acute COVID-19 infection with potential bacterial superinfection, such as prescribing corticosteroids and antibiotics. These practice patterns raise concern related to the potential adverse effects of these treatments, which may directly contribute to endocrinologic complications, increased vulnerability to future infections, and antimicrobial resistance [36]. The potential negative impacts of unnecessary corticosteroid use on immune function are particularly relevant in Botswana where HIV is widespread, with an estimated 21% prevalence rate [37].

Equally concerning is the fact that these approaches appear to be utilised in place of appropriate evaluation and multidisciplinary management for PASC symptoms. For instance, no respondent indicated evaluating patients with chronic shortness of breath for either cardiac sequellae of COVID-19 infection or for potential pulmonary complications such as fibrosis or pulmonary embolism. In the absence of a clear case definition for PASC currently in use in Botswana, there is also a risk that HCWs might inadvertently attribute symptoms to PASC that are in fact related to other common pathologies. For instance, in a high-TB setting, chronic cough due to this infection could be misattributed to prior COVID-19 infection, placing patients and their communities at risk. Furthermore, survey respondents themselves indicated a clear desire for clinical decision-making support in diagnosing and managing PASC.

In parallel with uncertainty regarding accurate diagnosis of PASC in the absence of a standardized case definition, the findings regarding PASC management underscore the need for streamlined management pathways for common PASC symptoms. In the Botswana context, HCWs (particularly those working in health posts and primary care clinics) are trained and skilled in the application of standardized evaluation and treatment algorithms for common conditions. Given the lack of existing national guidelines for PASC evaluation and management, it is unsurprising that primary HCWs report managing potential PASC-related symptoms according to established practices for undifferentiated headache, shortness of breath, persistent cough, and fatigue. To the best of our knowledge, one training was conducted on long-COVID during 2022, sponsored by the Botswana Ministry of Health in conjunction with the University of Botswana and disseminated to interested HCWs across the country as a live, synchronous online event. Beyond this, the authors (as HCWs who worked in Kweneng District during the period the survey was conducted) are unaware of other trainings or campaigns specific to PASC that may have influenced participant responses.

### Multidisciplinary care pathways

Beyond a need for training on appropriate identification and management of PASC symptoms, survey results demonstrate near unanimous demand among Kweneng District HCWs for multidisciplinary treatment pathways for patients suffering from PASC. This is well aligned with global recommendations for comprehensive PASC management [38,39]. Parkin and colleagues emphasise that primary health care systems globally must adopt a comprehensive and integrated multidisciplinary model of healthcare delivery to deal with the increasing number of post-COVID-19 cases effectively and efficiently [40]. In Botswana, plans for such models of integrated care delivery for PASC are in development but no such facility exists currently. The results of this survey demonstrate the clinical imperative to develop centres for multidisciplinary PASC care, which could also function as educational hubs for training and dissemination of management algorithms. In the absence of such guidance and facilities, HCWs across Botswana must rely on clinical reasoning, often in isolated contexts.

### Future research

Although not explicitly explored in the survey, the expressed uncertainty regarding PASC management is likely due in part to the absence of a clear case definition for PASC currently in use in Botswana. This raises concerns, as indicated above, regarding the possible conflation of chronic symptoms of PASC with other disease entities prevalent in Botswana. In addition, the absence of a commonly agreed time criterion to differentiate acute COVID-19, persistent COVID-19 infection, and PASC in Botswana is an important gap and leads to confusion among HCWs in determining how best to manage presenting symptoms thought to be related to COVID-19. Given the high background prevalence rate of HIV, the known risk of persistent COVID-19 infection in people living with HIV [41,42], and the current lack of point-of-care tests for active COVID-19 infection in the government sector in Botswana, contextually appropriate criteria for defining PASC in this setting is even more imperative. Additionally, both retrospective and prospective surveillance should be conducted of patients previously admitted to hospitals across Botswana for acute COVID-19 care, to determine the rates of COVID-19 complications (including but not limited to PASC) in this population.

Findings from this study have been disseminated to district health care leadership across professional cadres, including Nursing, Medicine, and Public Health. This has spurred requests for further research on the direct impact of the COVID-19 pandemic on frontline HCWs. Stakeholders engaged in dissemination sessions, particularly primary HCWs, have voiced requests for development of case definitions and clinical management algorithms for PASC to be used within Kweneng District, linked to advocacy for further guidance from the national level.

## Conclusions

The present study investigated HCW perceptions of PASC burden among patients presenting at primary care facilities and secondary hospitals, frequently encountered PASC symptoms, and typical PASC treatment strategies in Kweneng District, Botswana. The results of the survey yielded initial data on the perceived burden of PASC in Botswana as reported by frontline HCWs in Kweneng District, addressing an important gap in the existing evidence base. Additionally, the results of the survey provided insights into current management practices among providers at various health system levels, demonstrating demand for algorithms for PASC management to guide appropriate evaluation, diagnosis, and treatment of this syndrome. Further research is urgently needed to determine the true prevalence of PASC in Botswana, coupled with development and dissemination of case definitions for persistent COVID-19 infection and PASC, supported by algorithms for and training on PASC screening, diagnosis, and multidisciplinary management.

### Limitations

The generalizability of this study is limited to the context of Botswana. The initial response rate to online surveys was very low. This was attributed to lack of internet connectivity at workplaces in Kweneng District (particularly in rural areas), coupled with high cost of data for personal use. It is also possible that the low online survey response rate was related to lack of interest and/or sensitization among potential respondents. The relatively high response rate to the paper-based surveys, however, argues against self-selection or lack of interest among the target population, as the majority of HCWs offered the opportunity to complete the survey did so. Of note, in the few instances where HCWs invited to complete the paper survey declined to participate, they indicated that this was related to lack of relevance to current practice. Specifically, HCWs employed as midwives or engaged in inpatient duties outside of admitting roles stated that screening for potential PASC-related symptoms was not part of their routine workflow. In light of this, the total eligible target population is likely significantly lower than initially estimated, as it includes midwives and other HCWs who do not routinely manage PASC-related symptoms. Due to transportation barriers, the paper-based questionnaires were distributed to a convenience sample of HCWs, i.e. to primary facilities within Molepolole, which may have introduced bias by further limiting input from HCWs in more remote parts of the district. This risk was mitigated by also distributing the survey to HCWs escorting patients referred to the district hospital; these HCWs represented facilities up to 251km in distance from the district hospital. Following completion of the current rollout of cost-free Wi-Fi connections at government health facilities nationwide, we are hopeful that online surveys may help to address these gaps in future.

The survey was distributed several months following the conclusion of the most severe wave of acute COVID-19 infections in Botswana (the Delta wave, in mid-2021). This time point was selected intentionally, to allow for enough time to elapse for HCW participants to have some distance from managing acute COVID-19 cases and to increase the likelihood that participants were actively interacting with patients with possible long-term sequellae of infection. That said, the survey instrument asked HCWs to rely solely on their memory of patients treated for potential PASC symptoms to capture perceived importance of this problem, without triangulation from objective data sources. This represents an important source of likely recall bias. This limitation will be mitigated in future with the anticipated release of data prospectively collected from patients with confirmed prior COVID-19 infection.

## Supporting information

S1 Data Survey data. (XLSX)

S1 Text Survey tool: Long COVID-19 survey for district health workers. (DOCX)

S2 Text PLOS Inclusivity in global research checklist. (DOCX)

## References

[1] World Health Organisation. COVID-19 Epidemiological Update 2024 [updated 19 January 2024. Available from: https://www.who.int/docs/default-source/coronaviruse/situation-reports/20240119_covid-19_epi_update-handover_163.pdf?sfvrsn=67416922_4&download=true.

[2] Thaweethai T, Jolley SE, Karlson EW, Levitan EB, Levy B, McComsey GA, et al. Development of a definition of postacute sequelae of SARS-CoV-2 infection. Jama. 2023;329(22):1934–46. doi: 10.1001/jama.2023.8823 37278994 PMC10214179

[3] Bell ML, Catalfamo CJ, Farland LV, Ernst KC, Jacobs ET, Klimentidis YC, et al. Post-acute sequelae of COVID-19 in a non-hospitalized cohort: Results from the Arizona CoVHORT. PLoS One. 2021;16(8):e0254347. doi: 10.1371/journal.pone.0254347 34347785 PMC8336814

[4] Huang Y, Pinto MD, Borelli JL, Mehrabadi MA, Abrihim H, Dutt N, et al. COVID Symptoms, Symptom Clusters, and Predictors for Becoming a Long-Hauler: Looking for Clarity in the Haze of the Pandemic. medRxiv. 2021. 10.1177/10547738221125632 PMC9510954 36154716

[5] Nalbandian A, Sehgal K, Gupta A, Madhavan MV, McGroder C, Stevens JS, et al. Post-acute COVID-19 syndrome. Nature medicine. 2021;27(4):601–15. doi: 10.1038/s41591-021-01283-z 33753937 PMC8893149

[6] Weerahandi H, Hochman KA, Simon E, Blaum C, Chodosh J, Duan E, et al. Post-discharge health status and symptoms in patients with severe COVID-19. Journal of general internal medicine. 2021;36:738–45. doi: 10.1007/s11606-020-06338-4 33443703 PMC7808113

[7] National Institute for Health and Care Excellence: Clinical Guidelines. COVID-19 rapid guideline: managing the long-term effects of COVID-19. London: National Institute for Health and Care Excellence (NICE). Copyright © NICE 2020. 2024. 33555768

[8] Sherif ZA, Gomez CR, Connors TJ, Henrich TJ, Reeves WB. Pathogenic mechanisms of post-acute sequelae of SARS-CoV-2 infection (PASC). Elife. 2023;12:e86002. doi: 10.7554/eLife.86002 36947108 PMC10032659

[9] Soriano JB, Murthy S, Marshall JC, Relan P, Diaz JV. A clinical case definition of post-COVID-19 condition by a Delphi consensus. The Lancet infectious diseases. 2022;22(4):e102–e7. doi: 10.1016/S1473-3099(21)00703-9 34951953 PMC8691845

[10] Bansal R, Gubbi S, Koch CA. COVID-19 and chronic fatigue syndrome: An endocrine perspective. Journal of Clinical & Translational Endocrinology. 2022;27:100284. doi: 10.1016/j.jcte.2021.100284 34877261 PMC8641402

[11] Hanson SW, Abbafati C, Aerts JG, Al-Aly Z, Ashbaugh C, Ballouz T, et al. Estimated global proportions of individuals with persistent fatigue, cognitive, and respiratory symptom clusters following symptomatic COVID-19 in 2020 and 2021. Jama. 2022;328(16):1604–15. doi: 10.1001/jama.2022.18931 36215063 PMC9552043

[12] Vivaldi G, Pfeffer PE, Talaei M, Basera TJ, Shaheen SO, Martineau AR. Long-term symptom profiles after COVID-19 vs other acute respiratory infections: an analysis of data from the COVIDENCE UK study. EClinicalMedicine. 2023;65. doi: 10.1016/j.eclinm.2023.102251 38106559 PMC10721552

[13] Nyasulu PS, Tamuzi JL, Erasmus RT. Burden, Causation, and Particularities of long-COVID in African populations: A rapid systematic review. IJID regions. 2023. doi: 10.1016/j.ijregi.2023.08.004 37674565 PMC10477483

[14] Ozonoff A, Jayavelu ND, Liu S, Melamed E, Milliren CE, Qi J, et al. Features of acute COVID-19 associated with post-acute sequelae of SARS-CoV-2 phenotypes: results from the IMPACC study. Nature Communications. 2024;15(1):216. doi: 10.1038/s41467-023-44090-5 38172101 PMC10764789

[15] Masisi M. State of the Nation Address 202. 2020.

[16] Worldometer. Botswana COVID—Coronavirus Statistics, [Available from: https://www.worldometers.info/coronavirus/country/botswana/.

[17] Statistics Botswana. 2022 [Available from: https://www.statsbots.org.bw/2022-population-and-housing-census-preliminary-results.

[18] Mendelsohn AS, Nath N, De Sá A, Von Pressentin KB. Two months follow-up of patients with non-critical COVID-19 in Cape Town, South Africa. South African family practice. 2022;64(1). doi: 10.4102/safp.v64i1.5429 35144461 PMC8905322

[19] Zulu JE, Banda D, Hines JZ, Luchembe M, Sivile S, Siwingwa M, et al. Two-month follow-up of persons with SARS-CoV-2 infection-Zambia, September 2020: a cohort study. Pan African Medical Journal. 2022;41(1). doi: 10.11604/pamj.2022.41.26.30721 35291364 PMC8895565

[20] Osikomaiya B, Erinoso O, Wright KO, Odusola AO, Thomas B, Adeyemi O, et al. ’Long COVID’: persistent COVID-19 symptoms in survivors managed in Lagos State, Nigeria. BMC Infect Dis. 2021;21(1):304. doi: 10.1186/s12879-020-05716-x 33765941 PMC7993075

[21] El Otmani H, Nabili S, Berrada M, Bellakhdar S, El Moutawakil B, Abdoh Rafai M. Prevalence, characteristics and risk factors in a Moroccan cohort of Long-Covid-19. Neurological Sciences. 2022;43(9):5175–80. doi: 10.1007/s10072-022-06138-0 35614173 PMC9132567

[22] Jassat W, Mudara C, Vika C, Welch R, Arendse T, Dryden M, et al. A cohort study of post-COVID-19 condition across the Beta, Delta, and Omicron waves in South Africa: 6-month follow-up of hospitalized and nonhospitalized participants. International Journal of Infectious Diseases. 2023;128:102–11. doi: 10.1016/j.ijid.2022.12.036 36587841 PMC9800016

[23] Cabrera Martimbianco AL, Pacheco RL, Bagattini Â M, Riera R. Frequency, signs and symptoms, and criteria adopted for long COVID-19: A systematic review. Int J Clin Pract. 2021;75(10):e14357. doi: 10.1111/ijcp.14357 33977626 PMC8236920

[24] Yoo SM, Liu TC, Motwani Y, Sim MS, Viswanathan N, Samras N, et al. Factors associated with post-acute sequelae of SARS-CoV-2 (PASC) after diagnosis of symptomatic COVID-19 in the inpatient and outpatient setting in a diverse cohort. Journal of general internal medicine. 2022;37(8):1988–95. doi: 10.1007/s11606-022-07523-3 35391623 PMC8989256

[25] Crankson S, Pokhrel S, Anokye NK. Determinants of COVID-19-Related Length of Hospital Stays and Long COVID in Ghana: A Cross-Sectional Analysis. Int J Environ Res Public Health. 2022;19(1). doi: 10.3390/ijerph19010527 35010786 PMC8744866

[26] Karuna S, Gallardo-Cartagena JA, Theodore D, Hunidzarira P, Montenegro-Idrogo J, Hu J, et al. Post-COVID symptom profiles and duration in a global convalescent COVID-19 observational cohort: Correlations with demographics, medical history, acute COVID-19 severity and global region. J Glob Health. 2023;13:06020. doi: 10.7189/jogh.13.06020 37352144 PMC10289480

[27] Botswana. n.d [Available from: https://www.google.com/url?q = https://www.healthdata.org/research-analysis/health-by-location/profiles/botswana&sa=D&source=docs&ust=1713855387995852&usg=AOvVaw0wgFC7cUPpYlJc2deDiMnT.

[28] La Grange M. Prevalence of occupational lung disease among Botswana men formerly employed in the South African mining industry. Occupational and environmental medicine. 1998;55(8):577. doi: 10.1136/oem.55.8.577 9849548 PMC1757628

[29] World Health Organisation. Global tuberculosis report 2021 World Health [Available from: Organizationhttps://www.who.int/teams/global-tuberculosis-programme/data.

[30] Pretorius E, Vlok M, Venter C, Bezuidenhout JA, Laubscher GJ, Steenkamp J, et al. Persistent clotting protein pathology in Long COVID/Post-Acute Sequelae of COVID-19 (PASC) is accompanied by increased levels of antiplasmin. Cardiovascular diabetology. 2021;20:1–18. 34425843 10.1186/s12933-021-01359-7 PMC8381139

[31] Chelly S, Rouis S, Ezzi O, Ammar A, Fitouri S, Soua A, et al. Symptoms and risk factors for long COVID in Tunisian population. BMC Health Services Research. 2023;23(1):487. doi: 10.1186/s12913-023-09463-y 37189141 PMC10184965

[32] Frallonardo L, Segala FV, Chhaganlal KD, Yelshazly M, Novara R, Cotugno S, et al. Incidence and burden of long COVID in Africa: a systematic review and meta-analysis. Scientific Reports. 2023;13(1):21482. doi: 10.1038/s41598-023-48258-3 38057338 PMC10700349

[33] Nkomazana O, Phaladze N, Peersman W, Willcox M, Mash R. Human resources for health in Botswana: the results of in-country database and reports analysis. African Journal of Primary Health Care and Family Medicine. 2014;6(1):1–8. doi: 10.4102/phcfm.v6i1.716 26245420 PMC4564932

[34] Rodrigues AN, Dias ARN, Paranhos ACM, Silva CC, Bastos TdR, Brito BBd, et al. Headache in long COVID as disabling condition: a clinical approach. Frontiers in Neurology. 2023;14:1149294. doi: 10.3389/fneur.2023.1149294 37034080 PMC10076861

[35] Fujita K, Otsuka Y, Sunada N, Honda H, Tokumasu K, Nakano Y, et al. Manifestation of headache affecting quality of life in long COVID patients. Journal of Clinical Medicine. 2023;12(10):3533. doi: 10.3390/jcm12103533 37240639 PMC10219375

[36] Wallis RS. Corticosteroids and HIV infection: a review of experience. Current Opinion in HIV and AIDS. 2007;2(3):213–8. doi: 10.1097/COH.0b013e3280eec766 19372889

[37] UNAIDS. Country progress report—Botswana 2020 [Available from: https://www.unaids.org/sites/default/files/country/documents/BWA_2020_countryreport.pdf.]

[38] Munipalli B, Seim L, Dawson NL, Knight D, Dabrh AMA. Post-acute sequelae of COVID-19 (PASC): a meta-narrative review of pathophysiology, prevalence, and management. SN Compr Clin Med. 2022;4(1):90. doi: 10.1007/s42399-022-01167-4 35402784 PMC8977184

[39] Centers for Disease Control and Prevention. Post-COVID Conditions: Information for Healthcare Provider [updated February 6, 2024 Available from: https://www.cdc.gov/covid/hcp/clinical-overview/?CDC_AAref_Val=https://www.cdc.gov/coronavirus/2019-ncov/hcp/clinical-care/post-covid-conditions.html.]

[40] Parkin A, Davison J, Tarrant R, Ross D, Halpin S, Simms A, et al. A multidisciplinary NHS COVID-19 service to manage post-COVID-19 syndrome in the community. Journal of primary care & community health. 2021;12:21501327211010994. doi: 10.1177/21501327211010994 33880955 PMC8064663

[41] Yendewa GA, Perez JA, Patil N, McComsey GA. Associations between post-acute sequelae of SARS-CoV-2, COVID-19 vaccination and HIV infection: a United States cohort study. Frontiers in Immunology. 2024;15:1297195. doi: 10.3389/fimmu.2024.1297195 38318191 PMC10838972

[42] Yendewa GA, Perez JA, Schlick K, Tribout H, McComsey GA, editors. Clinical features and outcomes of coronavirus disease 2019 among people with human immunodeficiency virus in the United States: a multicenter study from a large global health research network (TriNetX). Open forum infectious diseases; 2021: Oxford University Press US. 10.1093/ofid/ofab272 PMC8244788 34435074

