# Supplementary material for: Perceptions of prevalence and management of post-acute sequelae of SARS-CoV-2 (PASC) infection among healthcare workers in Kweneng District, Botswana: Report of a district-wide survey
Source: PLOS Glob Public Health. 2024 Nov 27;4(11):e0003865. doi: 10.1371/journal.pgph.0003865 (PMC11602055; doi:10.1371/journal.pgph.0003865)
Supplement: S1 Text — (DOCX) [file pgph.0003865.s002.docx]

**Long COVID-19 survey for district health workers**

By proceeding with this survey, you indicate your consent to participate in the study as described above.**Tick/ check the appropriate response**

Do you agree to participate in this study?

|  | Yes, I agree to participate in this study |
| --- | --- |
|  | No, I do not wish to participate in this study |

What is your primary site of work?

|  | Scottish Livingstone Hospital |
| --- | --- |
|  | Thamaga Hospital |
|  | Primary clinic in Kweneng District |
|  | Health post in Kweneng District |
|  | Other: |

What is your professional designation?

|  | Nurse |
| --- | --- |
|  | Doctor |
|  | Specialist |
|  | Administrator |
|  | Other |

What is your age?

|  | 18-20 |
| --- | --- |
|  | 21-30 |
|  | 31-40 |
|  | 41-50 |
|  | 51-60 |
|  | 61-70 |
|  | Other: |

What is your gender?

|  | Female |
| --- | --- |
|  | Male |
|  | Prefer not to say |

How many COVID-19 vaccines have you administered in the past month, if any? You may provide an approximation.

|  |
| --- |

Which vaccine(s) have you administered in the past month, if any? Select all that apply.

|  | Sinovac or Sinopharm |
| --- | --- |
|  | AstraZeneca or Covishield |
|  | Johnson & Johnson |
|  | Pfizer |
|  | Moderna |
|  | Not applicable - I have not administered any of these vaccines |
|  | Other |
|  | Add option |

**Long COVID in your health care facility**

**The following questions ask about symptoms of long COVID and what you have noticed about patients who present for care for these symptoms. As you answer these questions, please think about all of the patients you have seen over the past *month* who came to your clinic.**

How many patients per week do you treat for symptoms of long COVID? You may provide an approximation

|  | 0-5 patients |
| --- | --- |
|  | 6-10 patients |
|  | 11-15 patients |
|  | 15-20 patients |
|  | More than 20 patients |

What percentage of the patients you see each week are seeking treatment for long COVID symptoms?

|  | Less than 10% of patients |
| --- | --- |
|  | 11% - 25% of patients |
|  | 26% - 50% of patients |
|  | 51% - 75% of patients |
|  | More than 75% of patients |
|  | Unsure |

How long after COVID-19 infection are these patients presenting?

|  | Less than 1 month |
| --- | --- |
|  | 1-2 months |
|  | 2-3 months |
|  | 3-6 months |
|  | Greater than 6 months |
|  | It varies |
|  | unsure |

In patients who present more than 4 weeks after COVID-19 infection with persistent symptoms, which of the following symptoms do you see most often? Please select the 5 most commonly seen symptoms.

|  | Persistent cough |
| --- | --- |
|  | Fever |
|  | Shortness of breath |
|  | Loss of taste/smell |
|  | Fatigue |
|  | Muscle or body aches |
|  | Headache |
|  | Sore throat |
|  | Congestion or runny nose |
|  | Nausea and/or vomiting |
|  | Diarrhea |
|  | Joint pain |
|  | Palpitations |
|  | Skin rash |
|  | Myalgia and weakness |
|  | Hair loss |
|  | Impaired balance and gait |
|  | Memory loss/difficulty concentrating |
|  | Other: |

Using the symptoms that you selected in the previous question, rank these symptoms in order of how often you see each symptom, from most common to least common (first = most

Your answer

1.

2.

3.

4.

5.

**Sick notes/leave**

The following questions ask about patients seeking sick notes/leave for COVID-19 related complications. Please think about all of the patients you have seen over the last week for COVID-19 related complications as you answer these questions

Have you noticed an increase in the number of sick notes/eave you are writing over the last year?

|  | Yes |
| --- | --- |
|  | No |
|  | I'm not sure |

How many times per week are you writing a sick note/eave for a patient due to COVID-19 related complications?

Your answer

How many days off of work do you give for a COVID-19 related complication? If you give different numbers of days for specific symptoms, please describe.

Your answer

Have most of the patients who are receiving sick notes for COVID-19 associated complications received a sick note for these symptoms before?

|  | Yes |
| --- | --- |
|  | No |
|  | I'm not sure |

**Treatment of long COVID symptoms**

The following questions ask about the treatments you currently recommend for certain COVID-19 related complications. Please answer the questions concerning any symptom you currently treat or have treated in the past.

How do you treat persistent cough due to long COVID?

Your answer

How do you treat persistent shortness of breath due to long COVID?

Your answer

How do you treat chronic headache due to long COVID?

Your answer

How do you treat intermittent chest pain due to long COVID?

Your answer

How do you treat fatigue due to long COVID?

Your answer

If any of the symptoms that you see most commonly are not listed above, please describe how you treat those symptoms below.

Your answer

If your clinic held a training/informative session for treatment of long COVID symptoms, would you be interested in attending?

|  | Yes |
| --- | --- |
|  | No |
|  | Maybe |

If your clinic included options for physiotherapy and/or occupational therapy services, would you consider referring patients with COVID-19 related complications to those services?

|  | Yes |
| --- | --- |
|  | No |
|  | Maybe |

If your clinic included options for mental health services, would you consider referring patients with COVID-19 related complications to those services?

|  | Yes |
| --- | --- |
|  | No |
|  | Maybe |

**Further information**

If you are interested in sharing more information about your experiences with long COVID symptoms, please call this number: -----. Thank you for taking the time to complete this survey - your help is appreciated!
